# Supplementary material for: Effect of Curcumin Plus Piperine on Redox Imbalance, Fecal Calprotectin and Cytokine Levels in Inflammatory Bowel Disease Patients: A Randomized, Double-Blind, Placebo-Controlled Clinical Trial
Source: Pharmaceuticals (Basel). 2024 Jun 28;17(7):849. doi: 10.3390/ph17070849 (PMC11279814; doi:10.3390/ph17070849)
Supplement: Supplementary file 1 [file pharmaceuticals-17-00849-s001.zip › pharmaceuticals-3006527-supplementary.pdf]

Table S1: Gastrointestinal complaints of patients with inflammatory bowel disease according to the treatment group: baseline data (T1) and endline data (T2).

		Total T1	Total T2	Group						GEE p (OR; 95% CI)
				Placebo		Curcumin		Curcumin + Piperine		
				T1 n=19	T2 n=14	T1 n=20	T2 n=20	T1 n=19	T2 n=17	
Pyrosis	No	34 (58.6)	39 (76.5)	13 (68.4)	13 (92.9)	13 (65.0)	17 (85.0)	8 (42.1)	9 (52.9)	0.737 (0.808; 0.234 – 2.791)#
	Yes	24 (41.4)	12 (23.5)	6 (31.6)	1 (7.1)	7 (35.0)	3 (15.0)	11 (57.9)	8 (47.1)	0.016 (4.151; 1.309 – 13.169)##
Nausea	No	40 (69.0)	42 (82.4)	13 (68.4)	13 (92.9)	16 (80.0)	16 (80.0)	11 (57.9)	13 (76.5)	0.909 (1.077; 0.304 – 3.816)#
	Yes	18 (31.0)	9 (17.6)	6 (31.6)	1 (7.1)	4 (20.0)	4 (20.0)	8 (42.1)	4 (23.5)	0.279 (0.538; 0.176 – 1.651)##
Vomiting	No	57 (98.3)	49 (96.1)	19 (100.0)	14 (100.0)	19 (95.0)	20 (100.0)	19 (100.0)	15 (88.2)	unavailable
	Yes	1 (1.7)	2 (3.9)	0 (0.0)	0 (0.0)	1 (5.0)	0 (0.0)	0 (0.0)	2 (11.8)	
Regurgitation	No	43 (74.1)	42 (82.4)	14 (73.7)	13 (92.8)	15 (75.0)	18 (90.0)	14 (73.7)	11 (64.7)	0.940 (1.048; 0.310 – 3.541)#
	Yes	15 (25.9)	9 (17.6)	5 (26.3)	1 (7.1)	5 (25.0)	2 (10.0)	5 (26.3)	6 (35.3)	0.279 (0.505; 0.146 – 1.741)##
Abdominal pain	No	29 (50.0)	41 (80.4)	10 (52.6)	12 (85.7)	12 (60.0)	17 (85.0)	7 (36.8)	12 (70.6)	0.597 (1.318; 0.473 – 3.670)#
	Yes	29 (50.0)	10 (19.6)	9 (47.4)	2 (14.3)	8 (40.0)	3 (15.0)	12 (63.2)	5 (29.4)	0.255 (0.559;0.205 – 1.522)##
Rectal bleeding	No	45 (77.6)	48 (94.1)	14 (73.7)	16 (84.2)	15 (75.0)	19 (95.0)	16 (84.2)	16 (94.1)	0.745

		Total T1	Total T2	Group						GEE p (OR; 95% CI)
				Placebo		Curcumin		Curcumin + Piperine		
				T1 n=19	T2 n=14	T1 n=20	T2 n=20	T1 n=19	T2 n=17	
										(1.259; 0.314 – 5.057)[#]
	Yes	13 (22.4)	3 (5.9)	5 (26.3)	3 (15.8)	5 (25.0)	1 (5.0)	3 (15.8)	1 (5.9)	0.428<br>(1.778; 0.429 – 7.370)[##]
Diarrhea	No	32 (55.2)	32 (62.7)	9 (47.4)	7 (50.0)	12 (60.0)	13 (65.0)	11 (57.9)	12 (70.6)	0.250<br>(1.771; 0.668 – 4.691)[#]
	Yes	26 (44.8)	19 (37.6)	10 (52.6)	7 (50.0)	8 (40.0)	7 (35.0)	8 (42.1)	5 (29.4)	0.258<br>(1.880; 0.630 – 5.609)[##]
Flatus	No	22 (37.9)	29 (56.9)	6 (31.6)	9 (64.3)	6 (30.0)	10 (50.0)	10 (52.6)	10 (58.8)	0.684<br>(0.800; 0.273 – 2.342)[#]
	Yes	36 (62.1)	22 (43.1)	13 (68.4)	5 (35.7)	14 (70.0)	10 (50.0)	9 (47.4)	7 (41.2)	0.471<br>(1.500; 0.498 – 4.514)[##]
Abdominal distention	No	28 (48.3)	39 (76.5)	8 (42.1)	12 (85.7)	10 (50.0)	16 (80.0)	10 (52.6)	11 (64.7)	0.711<br>(1.207; 0.446 – 3.264)[#]
	Yes	30 (51.7)	12 (23.5)	11 (57.9)	2 (14.3)	10 (50.0)	4 (20.0)	9 (47.4)	6 (35.3)	0.864<br>(0.910; 0.309 – 2.682)[##]

Legend: Data expressed as n (%); GEE = generalized estimating equations - group vs. time interaction, adjusted by sex, age and inflammatory bowel disease type; # = placebo group vs. *Curcuma longa* + piperine group; ## = placebo group vs. *Curcuma longa* group (absence of treatment was used as a reference).
